# The associations between stunting and wasting at 12 months of age and developmental milestones delays in a cohort of Cambodian children

**DOI:** 10.1038/s41598-022-22861-2

**Published:** 2022-10-25

**Authors:** Marion Van Beekum, Jacques Berger, Judit Van Geystelen, Gabriela Hondru, Somphos Vicheth Som, Chan Theary, Arnaud Laillou, Etienne Poirot, Kirsten A. Bork, Frank T. Wieringa, Sonia Fortin

**Affiliations:** 1 UMR Qualisud, Université Montpellier, Université Avignon, CIRAD, Institut Agro, IRD, Université de la Réunion, 911 Avenue Agropolis, 34 394 Montpellier Cedex, France; 2 Institut de Recherche pour le Développement (IRD), Montpellier, France; 3 Department of Child Survival and Development, United Nations Children’s Fund Cambodia, Phnom Penh, Cambodia; 4 Department of Health Sciences, Section Infectious Diseases, Vrije University, Amsterdam, The Netherlands; 5 Reproductive and Child Health Alliance (RACHA), Phnom Pen, Cambodia

**Keywords:** Malnutrition, Epidemiology, Paediatrics

## Abstract

Worldwide, over 250 million children under 5 years do not reach their developmental potential due to several causes, including malnutrition. In Cambodia, the prevalence of stunting and wasting among children remains high. This prospective cohort study aimed to assess acquisition of motor and cognitive developmental milestones in early childhood and their associations with stunting and wasting. Children aged from 0 to 24 months were recruited from three provinces in Cambodia and followed up to seven times from March 2016 to June 2019, until their 5 years. Data collection included anthropometry and developmental milestones. Seven motor and seven cognitive milestones were evaluated using the Cambodian Development Milestone Assessment Tool. Associations were assessed with parametric survival models. Hazard ratios (HR) below 1 stood for lower probabilities for achieving developmental milestones. Data were available for 7394 children. At 12 months, the prevalence of stunting and wasting were 23.7% and 9.6% respectively. Both were consistently associated with delays in most motor and cognitive milestones. Stunting was strongly associated with delays in gross motor milestones (HR < 0.85; *p* < 0.001). Wasting was more strongly associated with delays in fine motor development and most cognitive milestones (HR < 0.75; *p* < 0.001). Promoting nutritional programs in the first 1000 days to prevent malnutrition is essential to further the optimal growth and motor and cognitive development of Cambodian children.

## Introduction

Reducing the prevalence of malnutrition among children under 5 years of age still represents a global health priority for most low- and middle-income countries (LMICs). Globally, one in three children under five suffers from malnutrition, and two-thirds of them live in Asia^1^. In Cambodia, malnutrition is a real public health threat with 32.4% of children under 5 years of age being stunted and 9.7% wasted^2^. The Cambodian government is part of the Scaling Up Nutrition (SUN) network, and has committed itself to the Sustainable Development Goals including eradication of hunger and food insecurity^3^.

There is growing evidence of associations between linear growth retardation (leading to stunting and wasting) early in life and later sub-optimal health and educational performance^4–7^. During the first 1000 days of life, brain development is rapid and dynamic, and this period is important for establishing cognitive capacities^8–10^. Malnutrition can affect child development by impacting neurogenesis and myelination, thereby impairing cognitive and motor skills in infants^11^. Regardless of underlying pathogeneses, much more research is needed to understand the early origins, genetics and environmental influences leading to motor and cognitive developmental impairments.

Child development is usually assessed according to childhood developmental milestones, i.e. essential skills that define how a child functions in its environment (socially, physically and intellectually). There are many tools available to assess child development, but none of them is universally accepted^12^. Most tools use a "cut-off age", beyond which a lack of acquisition marks a developmental delay. One of the earliest tools was the Denver Developmental Screening Test (DDST), introduced in 1967, consisting of over 100 observations, grouped into four categories: gross motor skills (e.g., head control, standing), fine motor skills (e.g., grasping, scribbling), language (e.g., laughing, imitating sounds), and sociability (e.g., smiling, participating in games)^13^. To accurately assess child development, culturally appropriate screening tools must be used, as culture influences child environments^14,15^. Tools such as the DDST (or its revised version, the DDST II), may not be valid in all cultures as habits, traditions and development models vary across countries^16^. For example, most Cambodian children do not use pencil and paper until at least 5–6 years of age, whereas most of the DDST II fine motor tests for 3–5 year olds rely on drawing.

Recently, a reference developmental milestones chart, the "Cambodian Developmental Milestones Assessment Tool” (cDMAT) has been developed for Cambodian children, aged 0–83 months^17^. Key observations were selected from the DDST II, but modifications were made to adapt to the Cambodian culture. For example, "wave bye-bye" has been changed to "play chab chab" (gesture taught to Cambodian infants) or "play with a lotus seed" has been added. The cDMAT is composed of 140 developmental milestones (32 gross motor, 37 fine motor, 33 language and 38 social).

The aim of the present study was to assess a large cohort of Cambodian children under 5 years of age with regards to their acquisition of motor and cognitive milestones, using the cDMAT and to investigate whether delays in acquisition of these milestones are associated with malnutrition, defined as stunting and wasting (Height-for-Age and Weight-for-Age scores below the 2.5th percentile of the WHO standards).

## Methodology

### Study area and design

The MyHealth study was a prospective cohort study, conducted from March 2016 to January 2020 in six districts in three provinces in Cambodia: Phnom Penh (the capital, urban environment; 1 district), Kratie (around 200 km to the northeast of Phnom Penh, a mainly rural environment surrounding the Mekong River; 2 districts) and Ratanakiri (400 km to the northeast of Phnom Penh, a hilly rural environment; 3 districts). The protocol included collection of longitudinal data on health and nutritional status of young children^18^.

The minimum sample size was set at 1200 children per province to detect a difference in prevalence of stunting of 6% with a precision of 3%, an *α*-type error of 5%, a statistical power of 80% and a margin of 20% to account for loss to follow up and incomplete data^18^.

Women (pregnant or lactating) and children aged under 2 years of age were enrolled from March 2016 to August 2017. In addition, children born during the study from women participating in the MyHealth study were also included, and from august 2017 onwards, also children up to 5 years of age living in the study districts. Village health support groups and local midwives listed all pregnant women and children under the age of 5 years in the study areas to facilitate the recruitment process. Eligible infants and children were invited to participate in the MyHealth study until the minimum sample size was reached.

**Figure 1:**
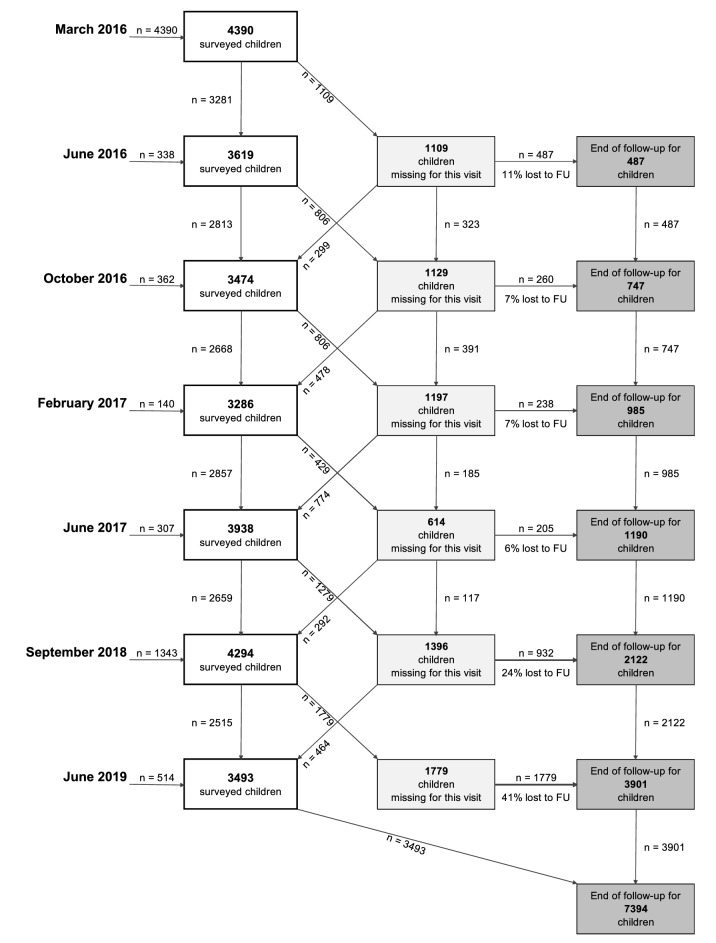
Flow chart of children included in the cohort analyses. *FU* Follow-up.

Recruited children were surveyed from their recruitment up to January 2020 with three visits in 2016, two visits in 2017 and one visit in 2018, 2019 and 2020 respectively. Sociodemographic and economic characteristics were collected at enrolment. At each timepoint, anthropometric and developmental measurements were collected. A longer follow-up period between visits in later years was chosen as motor and cognitive development changes more slowly at older ages. Data from children over 5 years were excluded from the present analysis. As most children were older than 5 years in the 2020 follow-up, only data collected up until June 2019 were used for the current analysis. From 2016 to 2017, between 6 and 11% of children were lost to follow-up at each round, mainly due to moving, especially in Phnom Penh which is a very dynamic urban area, with many households depending on labour in the garment industry. The flow chart of children included in the cohort study is described in Fig. 1.

Ethical approval for the study was obtained from the Cambodia National Ethical Committee for Health Research. The study was conducted in accordance to the Declaration of Helsinki for the protection of human subjects involved in research including confidentiality of personal information. Informed consent was obtained from all participants, with consent obtained from parents or guardians for participating children.

### Measurement and data sources

#### Sociodemographic and economic characteristics

Sociodemographic and economic characteristics (SEC) included province, age, gender, mother education (divided in four groups: ‘no formal school education’, ‘primary’, ‘secondary’, ‘high school or university’) and the economic level of the household. Because of large differences in SEC between the provinces, economic level was assessed for each province separately, with an index constructed from multiple correspondence analyses of a set of SEC variables collected at baseline and comprising household assets, living conditions (ownership of computer, phone, generator, radio or TV, non-motorised vehicle, motorised vehicle, principal source of light, principal source of energy for cooking), and hygiene (principal source of water for drinking, type of latrines)^19^. The coordinate on the first axis of the correspondence analysis was interpreted as a summary indicator of the economic level, standardized and categorized in three tertiles (‘Poor’, ‘Median’, ‘Wealthy’) per province in subsequent analyses.

#### Anthropometric measurements

Anthropometric measurements were collected at each visit using standard methods and Z-scores calculated according to WHO recommendations (WHO, 2006). Height (or recumbent length when the child was below 2 years of age) was measured to the nearest millimetre (Unicef measurement boards), while weight was measured to the nearest 100 g (2-in-1 Seca^®^ electronic scale). Birth dates were collected from health cards. Anthropometric status was defined as “stunted” for children with a height-for-age Z-score (HAZ) < − 2 and “wasted” for children with a weight-for-height Z-score (WHZ) < − 2. HAZ and WHZ at 12 and 24 months of age were calculated using linear interpolation, as the two follow-up visits around 12 or 24 months were done in an interval of maximal 6 months.

#### Motor and cognitive milestones

Motor and cognitive milestones were assessed at each visit using the cDMAT (Cambodian Development Milestones Assessment tool). The creation of a specific developmental screening tool for Cambodian children began in 2012 under the name "Angkor Hospital Development Milestones Assessment Tool (AHC DMAT)"^20^. Adjustments have been made from the Denver II Screening Test (DDST II) in order to adapt it to the Cambodian context, as described in the introduction^21^. A specific section of the cDMAT questionnaire was used in the present study to assess the motor and cognitive skills of children^17^. Also, a different set of observations was assessed during the seven follow-up surveys, to account for age. As Zubler et al.^22^, we used the four developmental domains classification: motor, socio-emotional, language and cognitive. In the present study, we define milestones relating to motor skills as motor milestones and milestones relating to socio-emotional, linguistic and cognitive skills as cognitive milestones. In total, seven motor milestones (“*Bring things to mouth*”, “*Sitting*”, “*Eat with hands*”, “*Standing*”, “*Walking*”, “*Palmer grasp*” and “*Drink from a cup*”) and seven cognitive milestones (“*Exogeneous smile*”, “*Follow with eyes*”, “*React to sound*”, “*Say no with head*”, *“Follow simple instructions*”, “*Interact with others*”, “*Say few words*”) were assessed. All milestones were dichotomous and coded ‘1’ for achieved, ‘0’ for unachieved, based on the interviewer’s observations and evaluation after discussion with the caretaker on the same day as the anthropometric measurements were taken. To minimize subjective judgement, interviewers attended specific training sessions before each follow-up.

In the present study we define child development as the ability to achieve the developmental milestones.

### Data management and statistical analyses

Data were collected on tablets, using a KoBo Toolbox questionnaire, by an experienced and trained team. Data were pseudonymised and stored on a KoBo Toolbox server, with a limited access to the core team of researchers only. First, quality checks were performed at data entry on the same day as data collection. Additional data cleaning and data management were then performed using Microsoft Excel 2016 (Microsoft Corporation) and R version 4.1.0 (The R Foundation for Statistical Computing). Statistical analyses were performed using R version 4.1.0.

Except ages of achievement for motor and cognitive milestones described using the 25th, 50th, 75th, 90th and 95th percentiles, quantitative variables were expressed as means and qualitative variables as percentages. Confidence intervals (95% CI) for these estimates were also computed.

Crude associations were tested using parametric survival models with age at observation for each milestone as outcomes and anthropometric status at 12 months as predictors. Models were left-censored based on date of birth. Adjusted associations were tested using the same survival models while controlling for province, mother education and child gender. Due to the heterogeneity of the economic level across the three provinces, models were run with and without controlling for economic tertile. Hazard’s ratios (HR), 95% CIs, and p-values were obtained for each explanatory variable. The first type error level was set at 0.05. A HR significantly below 1 implies that malnutrition was associated with a later acquisition of milestones for children with stunting (or wasting) compared to children without stunting (or without wasting).

### Informed consent statement

Written informed consent was obtained from all participants involved in the study.

## Results

### Sociodemographic characteristics

From March 2016 to June 2019, a total of 7394 children under five were surveyed: 2149 from Phnom Penh, 2815 from Kratie, and 2430 from Ratanakiri (Table 1). Mean age at recruitment was 10.3 months, as compared to 28.1 months at the last follow-up. Each child was surveyed between one and seven times with a mean of 3.6 visits. Half of the children were girls, 43% of mothers had primary school level education, 25% of mothers had secondary school level education, while 20% of mothers had no formal education.

**Table 1:** Characteristics of children as a whole and by province.

		All			Phnom Penh			Kratie			Ratanakiri		
		n	Mean/%	(CI)	n	Mean/%	(CI)	n	Mean/%	(CI)	n	Mean/%	(CI)
**Sociodemographic and economic characteristics**													
Gender	Male	7394	49.81	(48.66–50.96)	2149	51.84	(49.70–53.97)	2815	49.70	(47.83–51.56)	2430	48.15	(46.15–50.16)
	Female		50.19	(49.04–51.34)		48.16	(46.03–50.30)		50.30	(48.44–52.17)		51.85	(49.84–53.85)
Province	Phnom Penh	7394	29.06	(28.03–30.12)	2149	100	(100–100)	2815	0	(0–0)	2430	0	(0–0)
	Kratie		38.07	(36.96–39.19)		0	(0–0)		100	(100–100)		0	(0–0)
	Ratanakiri		32.86	(31.80–33.95)		0	(0–0)		0	(0–0)		100	(100–100)
Mother education	No education	6769	19.50	(18.57–20.47)	1930	8.55	(7.36–9.91)	2621	12.06	(10.85–13.38)	2218	37.83	(35.81–39.89)
	Primary		43.42	(42.23–44.61)		37.98	(35.81–40.19)		51.09	(49.15–53.02)		39.09	(37.06–41.16)
	Secondary		25.13	(24.10–26.18)		32.64	(30.56–34.79)		27.20	(25.52–28.96)		16.14	(14.65–17.75)
	High school/university		11.95	(11.19–12.75)		20.83	(19.05–22.72)		9.65	(8.56–10.86)		6.94	(5.94–8.10)
Economic level	Poor	5005	33.89	(32.58–35.22)	1641	35.71	(33.40–38.09)	1823	33.02	(30.87–35.24)	1541	32.97	(30.63–35.39)
	Median		36.20	(34.87–37.56)		43.27	(40.86–45.71)		32.58	(30.44–34.80)		32.97	(30.63–35.39)
	Wealthy		29.91	(28.65–31.20)		21.02	(19.09–23.09)		34.39	(32.22–36.63)		34.07	(31.71–36.51)
**Anthropometry**													
Stunting at 12 months		3134	23.71	(22.24–25.24)	764	15.71	(13.24–18.53)	1304	21.47	(19.29–23.82)	1066	32.18	(29.39–35.09)
Stunting at 24 months		3464	37.53	(35.92–39.17)	829	26.54	(23.59–29.71)	1479	35.90	(33.46–38.42)	1156	47.49	(44.58–50.42)
Wasting at 12 months		3138	9.59	(8.60–10.69)	767	7.43	(5.72–9.58)	1303	10.05	(8.50–11.85)	1068	10.58	(8.83–12.62)
Wasting at 24 months		3455	9.15	(8.22–10.17)	830	5.54	(4.13–7.38)	1476	8.88	(7.50–10.47)	1149	12.10	(10.30–14.16)

*CI* confidence interval.

### Anthropometry

HAZ and WHZ were normally distributed at 12 and 24 months. Prevalence of stunting increased with age from 23.7% at 12 months to 37.5% at 24 months, whereas prevalence of wasting did not change (9.6% at 12 months and 9.2% at 24 months, Table 1). Anthropometric indicators were significantly lower in Ratanakiri and Kratie than in Phnom Penh. Indeed, at 12 months, 15.7% of children were stunted and 7.4% were wasted in Phnom Penh *vs*. respectively 32.2% and 10.6% in Ratanakiri, and 21.5% and 10.1% in Kratie.

### Motor and cognitive milestones

The first motor milestone to be achieved was “*Bring to mouth*” with a median age of achievement equal to 7 months whereas the last milestones to be achieved were “*Walking*” and “*Palmer grasp*” with median ages of achievement equal to 14 months (Supplementary Table 1). The first cognitive milestone to be achieved was “*Smile*” with a median age of achievement equal to 2 months whereas the last one was “*Say few words*” with a median age equal to 15 months.

### Crude associations of motor and cognitive milestones with socioeconomic characteristics

Ages at achievement for motor and cognitive milestones were not associated with gender, but differed across provinces for four of the seven motor milestones and three of the seven cognitive milestones (Supplementary Tables 2 and 3). Children from Phnom Penh achieved “*Eat with hands*”, “*Palmer grasp*”, “*Say few words*” and “*Interaction with others*” later than children from the other two provinces, but “*Standing*” and “*Follow simple instructions*” earlier. Children from Kratie were slower to stand, walk and follow simple instructions, whereas children from Ratanakiri were faster to walk, eat with hands, palmer grasp, say a few words and interact with others. Mother’s education was significantly associated with the age of achievement of the motor milestones “*Bring things to mouth*”, “*Eat with hands*” and “*Palmer grasp*” and all the cognitive milestones (*p* < 0.05), with children of mothers with higher education achieving milestones earlier. Economic levels were significantly associated with age at achievement for “*Sitting*”, “*Standing*”, “*Walking*”, “*Drink from a cup*”, “*Say no with head*” and “*Follow simple instructions*” (*p* < 0.02), with children from the poorest tertile achieving these milestones later than those from the median and wealthy tertiles.

### Crude and adjusted associations of motor and cognitive milestones with stunting and wasting

**Figure 2:**
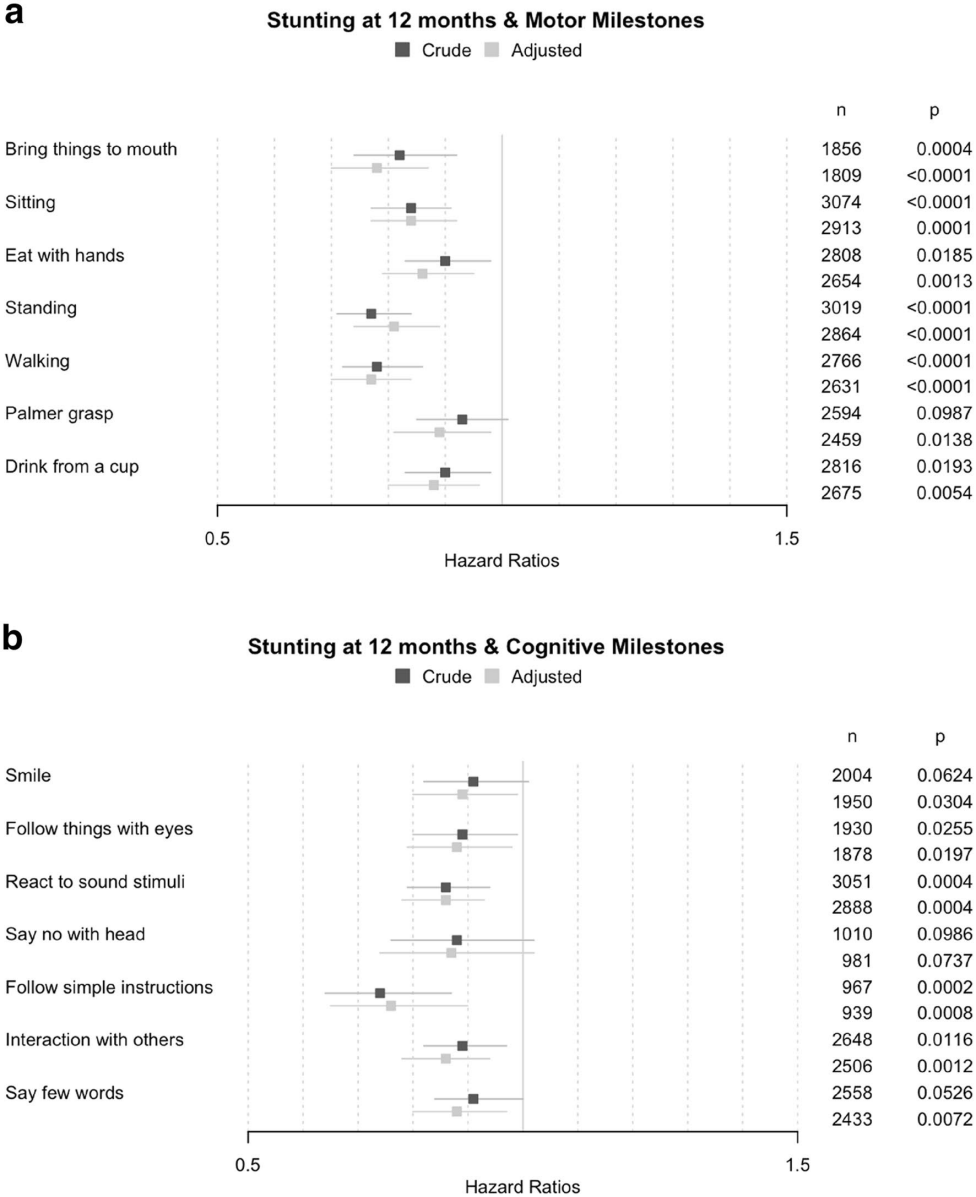

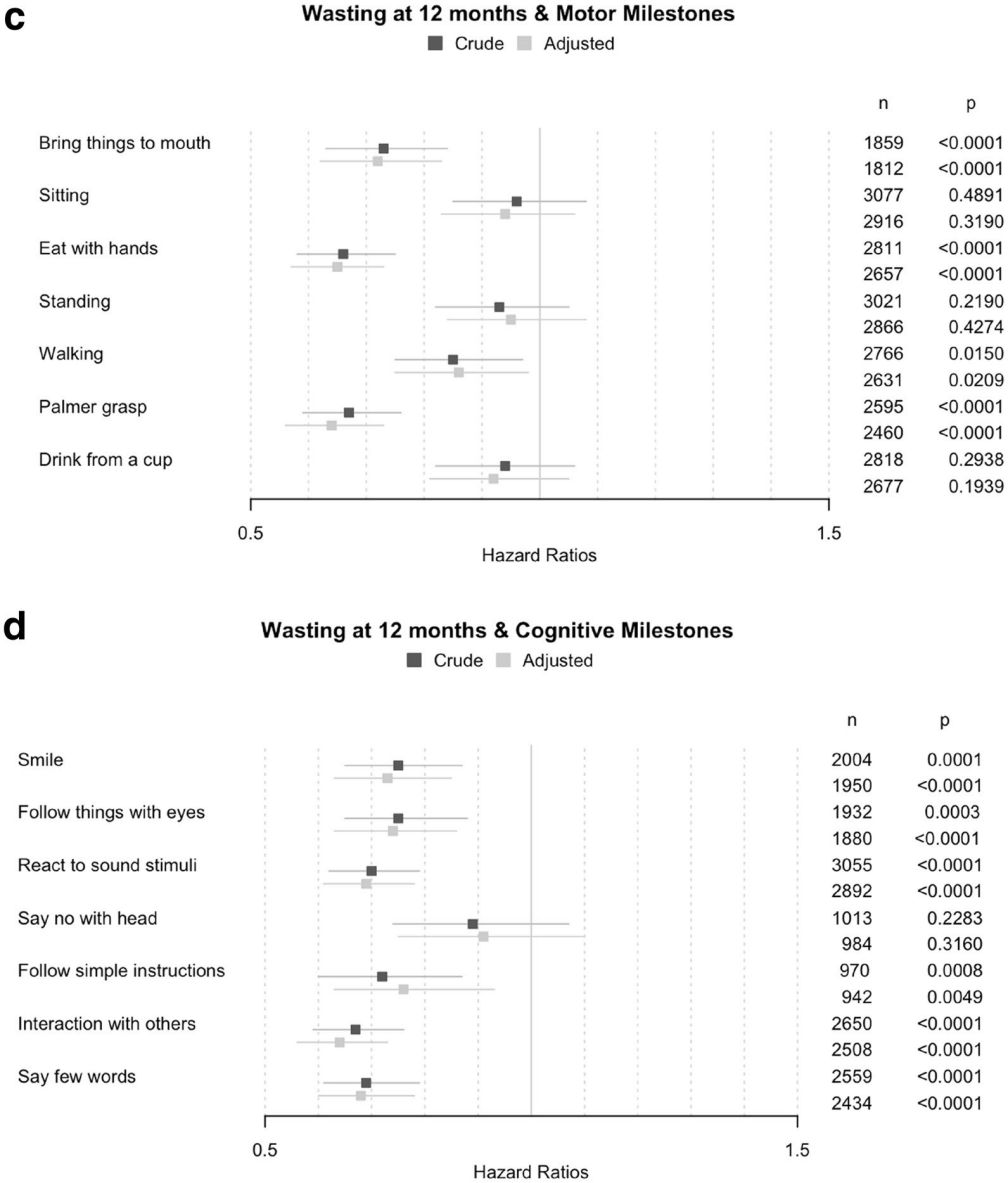
Associations between the ages for achieving motor and cognitive milestones and stunting and wasting at 12 months. Adjusted associations hazard ratios were controlled for province, mother education and child gender.

Children with stunting or wasting at 12 months displayed a tendency to achieve most of motor and cognitive milestones later than children without any stunting or wasting at 12 months (Supplementary Table 1). Unadjusted models showed that children with stunting at 12 months achieved six of the seven motor milestones later (HR from 0.77 to 0.90; *p* < 0.02) and four of the seven cognitive milestones later (HR from 0.74 to 0.89; *p* < 0.03), as compared to their counterparts without stunting (Fig. 2). After adjustment for province, maternal education and child gender, all associations between stunting and milestones except “*Say no with head*” became statistically significant. “*Follow simple instructions*”, “*Walking*” and “*Bring things to mouth*” were the milestones most delayed for children with stunting (HR equal to 0.76, 0.77 and 0.78, respectively; *p* < 0.001).

Unadjusted models showed that children with wasting at 12 months were significantly slower to achieve four of the seven motor milestones (HR from 0.67 to 0.85; *p* < 0.02) and six of the seven cognitive milestones (HR from 0.67 to 0.75; *p* < 0.001). After adjustment, these associations remained statistically significant. “*Palmer grasp*”, “*Interaction with others*” and “*Eat with hands*” were the milestones the most strongly associated with wasting (HR equal to 0.64, 0.64 and 0.65, respectively; *p* < 0.001).

Controlling for economic level in the models on stunting and wasting did not change any of the outcomes, but reduced the sample size because of missing data on household economic status. Repeating the models for stunting and wasting at 24 months gave similar results.

## Discussion

To our knowledge, this study is the first to combine repeated measures of anthropometry and child development during early childhood in Cambodia. We found strong associations between anthropometric status at 12 months and ages of achievement of most early developmental milestones. Stunting and wasting were consistently associated with delays in achieving motor and cognitive milestones in early childhood and significantly so for most of them (for 13 out of the 14 of milestones for stunting, and for 10 out of the 14 for wasting).

These associations found in Cambodia are consistent with previous findings in other countries^23–26^, especially in low and middle-income countries^23^. At school age, children’s cognitive performance is associated not only with concomitant anthropometric measures^24,26–29^ but also with linear growth during early childhood^7,30,31^. Before 2 years of age, child development is assessed mainly through motor performance and to a lesser extent through cognitive performance. However, several studies have also shown that global child development is associated with nutritional status in early childhood, in particular with low birth weight, and to a lesser extent with stunting and wasting^32–35^. Even though motor and cognitive development is fast throughout childhood, the first 1000 days are a critical period^11,23,36,37^. Our study, which focuses on what happens from birth, therefore responds particularly to the need to characterize this critical period of life.

We found that stunting and wasting at 12 months of age were both strongly associated with achieving motor and cognitive milestones, but with subtle differences. First, stunting was significantly associated with delays in achieving 13 out of the 14 measured milestones, but it was most strongly associated (HR < 0.85) with gross motor milestones (“*Sitting”*, “*Standing”* and “*Walking”*) and to a lesser extend (HR > 0.85) with fine motor (except 0.78 for “*Bring things to mouth*”) and cognitive milestones (except 0.76 for “*Follow simple instructions*”). Stunting was indifferently associated with early achieved milestones and later achieved milestones, suggesting long-lasting effects of malnutrition in utero and during the first year of life.

Associations between stunting and cognitive skills were already shown^7^ but there was no evidence for any association with motor and social development. Moreover, in a cross-sectional study conducted in 36–59-month-old children from four countries in South Asia (Bangladesh, Bhutan, Nepal and Pakistan), Kang et al.^38^ showed that stunting was negatively associated with cognitive development; however cognitive development was generally assessed through a composite score. Thus, our results confirm the associations highlighted in these studies on stunting and cognitive development, but show as well associations of stunting with motor development. Therefore, stunting at 12 months appears to be a good indicator of risk for delays in all domains of child development, even though it is likely that the nutritional insults took place before 12 months of age.

Second, wasting at 12 months was significantly associated with delays in achieving 10 out of the 14 measured milestones, and it was especially strongly associated with fine motor milestones (HR < 0.75 for “*Bring things to mouth”*, “*Eat with hands”* and “*Palmer grasp”*) and the later achieved cognitive milestones (HR < 0.70 for “*Say few words”* and “*Interaction with others”*). Wasting was also associated with other cognitive milestones (HR < 0.80 for “*Follow things with eyes”*, “*React to sound*”, “*Smile*” and “*Follow simple instructions*”) and to a lesser extent with “*Walking*” (HR = 0.86). Thus, our study offers new evidence that alongside stunting, wasting is also associated with all cognitive and motor early child development domains. Moreover beyond that, wasting can also have an impact later in life^39^.

It should be emphasized, and as argued by Leroy and Frongillo^40^, the strong statistical association between undernutrition and delay in cognitive development does not necessarily mean causality. Indeed, mothers of undernourished infants and young children might have caring practices which differ from those of mothers of well-nourished children, independently of socioeconomic status. It is well-known that cognitive stimulation has a positive impact on infant development^41^. Unfortunately, it is very challenging to document caring practices (e.g., time spent in interaction with child daily) with sufficient precision, thus no attempt was done to adjust for these factors. In the present case, it seems likely that poor dietary intakes^18^, leading to macro- and micronutrient deficiencies^42^, contribute to both growth faltering and sub-optimal child development in Cambodia. Besides nutrition, we also found associations of mother education and economic status with milestones achieved from birth to 5 years, showing the complexity of factors enabling (or hampering) early child development^43,44^.

It is well known that micronutrient status of the mother during pregnancy and of the child during early childhood directly impacts brain development^37^ and cognitive development. Some mechanisms involved are well documented in the literature. For example, iron-deficiency anemia is associated with poor cognitive and motor development and poorer mother-infant bonding^41,45,46^, and iodine and thiamine deficiencies are involved in brain development^47–49^. Unfortunately, we do not have data on biomarkers for micronutrient status in either mothers or children from the MyHealth study. However, national data on micronutrient status has shown that the prevalence of iron deficiency in mothers and children less than 24 months of age is not very high and close to 9% and 7% respectively, even if approximately 50% of the entire population are anaemic due to genetic disorders^42^. In contrast, > 50% of infants aged 6–12 months had urinary iodine concentrations regarded as deficient (< 100 µg/L), and at least 38% of infants had thiamine deficiency, using the most strict cut-off^50,51^. Recently, we showed a positive impact of thiamine supplementation of lactating Cambodian mothers on expressive and receptive language development of their infants at 6 months of age^52^. In children with stunting, the prevalence of iodine and thiamine deficiencies are likely to be higher, and have certainly contributed to delays in achieving motor and cognitive milestones.

Study strengths include the long follow-up duration (18 months on average), which allowed for survival analyses comparing probabilities of achievement for the 14 milestones according to anthropometric status and socio-economic groups. It also enabled the distinction between early versus later achieved skills, and between motor skills and cognitive skills. As shown in the literature, child development delays are already evident in the first year of life^23^, thus we focused the analysis on anthropometric status at 12 months rather than 24 months or later. However, anthropometrical status (stunting and wasting) at 24 months of age was similarly associated with most of the 14 milestones (data not shown, available on request). Another strength of the study is the use of a child development tool adapted and validated for Cambodian children. Indeed, we found estimated ages for motor and cognitive achievement milestones consistent with international standards found in the literature (Supplementary Table 3)^53–56^. Obviously, the cultural heterogeneity of the three provinces and the resulting mother and father behaviours influenced the use and results of the tool; however, the adjustment for provinces considered for the analyses allowed for rigorously assessing differences in developmental milestones achievement between children with stunting and children without stunting, and between children with wasting and children without wasting. It should also be noted that additional analyses, adjusted for the economic index, led to similar associations between anthropometric status and stages of development (Supplementary Tables 4 and 5). Unfortunately, the adapted tool also implies that child performance cannot be compared to standardized scores such as Bayley III or ASQ^57,58^.

Even though the underlying mechanisms of delayed child development have still to be explored, our study reinforces the notion that early stunting and early wasting are strongly associated with delays in all motor and cognitive development domains. Acknowledging the multiple factors that contribute to delays in motor and cognitive development as well as to stunting and wasting are essential when designing programs to improve motor and cognitive development. Supplementary longitudinal follow-ups could be useful to better describe developmental trajectories and the nature of delays, and to disambiguate between chronic disability and transient delays, enabling better defining of effective intervention programs.

Promoting nutritional programs in the first 1000 days to prevent malnutrition are essential to further not only optimal growth, but also motor and cognitive development of Cambodian children.

## Supplementary Information

Supplementary Table 1.

Supplementary Table 2.

Supplementary Table 3.

Supplementary Table 4.

Supplementary Table 5.

## Data Availability

The datasets generated and/or analysed during the current study are not publicly available as the study was conducted in a collaboration with UNICEF and the Ministry of Health, Cambodia, and data are property of the Ministry of Health, Cambodia, but are available from the corresponding author on reasonable request.
