# Supplementary material for: The associations between stunting and wasting at 12 months of age and developmental milestones delays in a cohort of Cambodian children
Source: Sci Rep. 2022 Oct 25;12:17859. doi: 10.1038/s41598-022-22861-2 (PMC9596435; doi:10.1038/s41598-022-22861-2)
Supplement: Supplementary file 1 — Supplementary Table 1. [file 41598_2022_22861_MOESM1_ESM.docx]

|  | MyHealth | | | MyHealth  Stunted children at 12 months | | | | | MyHealth  Wasted children at 12 months | | | | CDC^1^ | | Werner^2^ | | WHO motor^3^ | | Research MS comp. (2012)^4^ | | |  |
| --- | --- | --- | --- | --- | --- | --- | --- | --- | --- | --- | --- | --- | --- | --- | --- | --- | --- | --- | --- | --- | --- | --- |
|  | n | Percentiles  (in month of age) | | n | | Percentiles (in month of age) | | | n | | Percentiles (in month of age) | | |  | |  | |  | |  | | |
|  |  | 50% | 95% | |  | | 50% | 95% | |  | 50% | 95% | |  | |  | |  | |  | | |
| Motor milestones |  |  |  | |  | |  |  | |  |  |  | |  | |  | |  | |  | | |
| Bring things to mouth | 4172 | 7 | 10 | | 1856 | | 7 | 10 | | 1859 | 7 | 10 | |  | |  | |  | |  | | |
| Sitting | 6409 | 8 | 11 | | 3074 | | 8 | 11 | | 3077 | 9 | 12 | | [6 - 9] | | 5 | | 9 (3.8-9.32) | | < 6 | | |
| Eat with hands | 5422 | 9 | 13 | | 2808 | | 10 | 14 | | 2811 | 10 | 17 | |  | | [6 - 9] | |  | | < 18 | | |
| Standing | 6114 | 10 | 14 | | 3019 | | 11 | 15 | | 3021 | 11 | 13 | | [9 - 12] | | [9 - 12] | | 11.4 (4.8-11.4) | | < 12 | | |
| Walking | 5506 | 14 | 19 | | 2766 | | 14 | 22 | | 2766 | 14 | 20 | | [12 - 18] | | [12 - 18] | | 17.6 (8.2-17.6) | | < 18 | | |
| Palmer grasp | 4869 | 14 | 21 | | 2594 | | 15 | 21 | | 2595 | 14 | 23 | | [12 - 18] | | [9 - 12] | |  | | < 24 | | |
| Drink from a cup | 5641 | 13 | 18 | | 2816 | | 13 | 18 | | 2818 | 13 | 18 | |  | |  | |  | |  | | |
| Cognitive milestones |  |  |  | |  | |  |  | |  |  |  | |  | |  | |  | |  | | |
| Smile | 2826 | 2 | 5 | | 2004 | | 3 | 5 | | 2004 | 2 | 6 | |  | |  | |  | |  | | |
| Follow things with eyes | 2685 | 3 | 7 | | 1930 | | 4 | 7 | | 1932 | 3 | 7 | | [0 - 2] | | [1 - 2] | |  | | [0 - 2] | | |
| React to sound stimuli | 6458 | 3 | 7 | | 3051 | | 4 | 8 | | 3055 | 3 | 10 | | [2 - 4] | | [1 - 3] | |  | | [0 - 2] | | |
| Say no with head | 2902 | 13 | 18 | | 1010 | | 13 | 16 | | 1013 | 13 | 17 | | 18 | | [12 - 18] | |  | | < 24 | | |
| Follow simple instructions | 2706 | 14 | 19 | | 967 | | 13 | 16 | | 970 | 13 | 16 | |  | |  | |  | |  | | |
| Interaction with others | 5094 | 14 | 24 | | 2648 | | 16 | 23 | | 2650 | 15 | 24 | | > 12 | | > 12 | |  | | < 18 | | |
| Say few words | 4850 | 15 | 23 | | 2558 | | 16 | 23 | | 2559 | 16 | 23 | |  | |  | |  | |  | | |
| ^1^ CDC (2019) Important milestones, https://www.cdc.gov/ncbddd/actearly/milestones/milestones-2mo.html, last accessed Oct 4, 2019, US | | | | | | | | | | | | | | | | | | | | |  |  |
| ^2^ Werner D (2018) Disabled Village Children; A guide for community health workers, rehabilitation workers, and families, Hesperian Health Guides, 2nd edition, 9th printing, US | | | | | | | | | | | | | | | | | | | | |  |  |
| ^3^ WHO (2006) Motor Development Study: Windows of achievement for six gross motor development milestones, WHO Multicentre Growth Reference Study Group1, Acta Pædiatrica, Suppl 450: 86/95, SW | | | | | | | | | | | | | | | | | | | | |  |  |
| ^4^ Dosman CF, Andrews D, et al (2012) Evidence-based milestone ages as a framework for developmental surveillance, Paediatrics & Child Health Vol 17 No 10 p561-568, US | | | | | | | | | | | | | | | | | | | | |  |  |

**Supplementary table 1**: Ages for achieving development milestones
