# Supplementary material for: The associations between stunting and wasting at 12 months of age and developmental milestones delays in a cohort of Cambodian children
Source: Sci Rep. 2022 Oct 25;12:17859. doi: 10.1038/s41598-022-22861-2 (PMC9596435; doi:10.1038/s41598-022-22861-2)
Supplement: Supplementary file 2 — Supplementary Table 2. [file 41598_2022_22861_MOESM2_ESM.docx]

|  |  | Bring things to mouth | | | | | Sitting | | | | | | | | | Eat with hands | | | | | | Standing | | | | |
| --- | --- | --- | --- | --- | --- | --- | --- | --- | --- | --- | --- | --- | --- | --- | --- | --- | --- | --- | --- | --- | --- | --- | --- | --- | --- | --- |
|  |  | n | HR | CI | *p* | n | | | HR | | CI | | | *p* | n | | HR | | CI | | *p* | n | HR | | CI | *p* |
| Gender | Male | 4172 | 1 | - | 0.7420 | 6409 | | | 1 | | - | | | 0.8029 | 5422 | | 1 | | - | | 0.7152 | 6114 | 1 | | - | 0.0624 |
|  | Female |  | 1.01 | (0.95 - 1.07) |  |  | | | 0.99 | | (0.95 - 1.04) | | |  |  | | 1.01 | | (0.96 - 1.07) | |  |  | 0.95 | | (0.91 - 1.00) |  |
| Province | Phnom Penh | 4172 | 1 | - | 0.0532 | 6409 | | | 1 | | - | | | 0.6667 | 5422 | | 1 | | - | | 0.0016 | 6114 | 1 | | - | 0.0103 |
|  | Kratie |  | 1.07 | (1.00 - 1.16) |  |  | | | 1.01 | | (0.95 - 1.08) | | |  |  | | 1.12 | | (1.04 - 1.20) | |  |  | 0.92 | | (0.87 - 0.98) |  |
|  | Ratanakiri |  | 0.98 | (0.91 - 1.05) |  |  | | | 1.11 | | (1.04 - 1.18) | | |  |  | | 1.19 | | (1.10 - 1.27) | |  |  | 0.98 | | (0.92 - 1.05) |  |
| Mother education | No education | 4030 | 1 | - | 0.0151 | 6000 | | | 1 | | - | | | 0.8366 | 5062 | | 1 | | - | | 0.0040 | 5736 | 1 | | - | 0.3718 |
|  | Primary |  | 1.11 | (1.02 - 1.20) |  |  | | | 0.99 | | (0.93 - 1.06) | | |  |  | | 1.11 | | (1.04 - 1.20) | |  |  | 1.03 | | (0.96 - 1.11) |  |
|  | Secondary |  | 1.20 | (1.09 - 1.32) |  |  | | | 1.08 | | (1.00 - 1.17) | | |  |  | | 1.21 | | (1.12 - 1.32) | |  |  | 1.18 | | (1.09 - 1.28) |  |
|  | High School/  University |  | 1.11 | (1.00 - 1.24) |  |  | | | 1.09 | | (1.00 - 1.20) | | |  |  | | 1.12 | | (1.02 - 1.25) | |  |  | 1.21 | | (1.10 - 1.33) |  |
| Economic level | Poor | 4088 | 1 | - | 0.3763 | 4693 | | | 1 | | - | | | 0.0004 | 3762 | | 1 | | - | | 0.7588 | 4572 | 1 | | - | < 0.0001 |
|  | Median |  | 1.03 | (0.96 - 1.11) |  |  | | | 1.13 | | (1.06 - 1.21) | | |  |  | | 1.01 | | (0.94 - 1.09) | |  |  | 1.19 | | (1.11 - 1.27) |  |
|  | Wealthy |  | 1.08 | (0.99 - 1.17) |  |  | | | 1.17 | | (1.09 - 1.26) | | |  |  | | 1.17 | | (1.06 - 1.30) | |  |  | 1.25 | | (1.16 - 1.34) |  |
|  |  |  |  |  |  |  | | |  | |  | | |  |  | |  | |  | |  |  |  | |  |  |
|  |  | Walking | | | | | Drink from a cup | | | | | | | | | Palmer grasp | | | | | |  |  |  | |  |
|  |  | n | HR | CI | *p* | | | n | | HR | | CI | *p* | | | n | | HR | | CI | *p* |  |  | |  |  |
| Gender | Male | 5506 | 1 | - | 0.6137 | | | 5641 | | 1 | | - | 0.7909 | | | 4869 | | 1 | | - | 0.8059 |  |  | |  |  |
|  | Female |  | 0.99 | (0.94 - 1.04) |  | | |  | | 0.99 | | (0.94 - 1.05) |  | | |  | | 1.01 | | (0.95 - 1.07) |  |  |  | |  |  |
| Province | Phnom Penh | 5506 | 1 | - | 0.0012 | | | 5641 | | 1 | | - | 0.6898 | | | 4869 | | 1 | | - | 0.0008 |  |  | |  |  |
|  | Kratie |  | 0.90 | (0.84 - 0.96) |  | | |  | | 0.99 | | (0.92 - 1.05) |  | | |  | | 1.13 | | (1.05 - 1.22) |  |  |  | |  |  |
|  | Ratanakiri |  | 1.09 | (1.02 - 1.17) |  | | |  | | 1.16 | | (1.08 - 1.24) |  | | |  | | 1.22 | | (1.13 - 1.31) |  |  |  | |  |  |
| Mother education | No education | 5187 | 1 | - | 0.2754 | | | 5307 | | 1 | | - | 0.8578 | | | 4565 | | 1 | | - | 0.0129 |  |  | |  |  |
|  | Primary |  | 1.04 | (0.97 - 1.12) |  | | |  | | 1.01 | | (0.94 - 1.08) |  | | |  | | 1.10 | | (1.02 - 1.19) |  |  |  | |  |  |
|  | Secondary |  | 1.06 | (0.98 - 1.16) |  | | |  | | 1.04 | | (0.96 - 1.13) |  | | |  | | 1.18 | | (1.08 - 1.29) |  |  |  | |  |  |
|  | High School/  University |  | 1.26 | (1.14 - 1.39) |  | | |  | | 1.14 | | (1.03 - 1.25) |  | | |  | | 1.11 | | (1.00 - 1.24) |  |  |  | |  |  |
| Economic level | Poor | 4279 | 1 | - | < 0.0001 | | | 4335 | | 1 | | - | < 0.0001 | | | 3641 | | 1 | | - | 0.1748 |  |  | |  |  |
|  | Median |  | 1.27 | (1.18 - 1.36) |  | | |  | | 1.26 | | (1.17 - 1.35) |  | | |  | | 1.06 | | (0.98 - 1.14) |  |  |  | |  |  |
|  | Wealthy |  | 1.14 | (1.06 - 1.23) |  | | |  | | 1.49 | | (1.38 - 1.62) |  | | |  | | 1.23 | | (1.11 - 1.37) |  |  |  | |  |  |

HR: Hazard ratio, CI: Confidence Interval

**Supplementary table 2**: Crude associations between the ages for achieving motor milestones and sociodemographic and economic characteristics of children
