# Supplementary material for: The associations between stunting and wasting at 12 months of age and developmental milestones delays in a cohort of Cambodian children
Source: Sci Rep. 2022 Oct 25;12:17859. doi: 10.1038/s41598-022-22861-2 (PMC9596435; doi:10.1038/s41598-022-22861-2)
Supplement: Supplementary file 3 — Supplementary Table 3. [file 41598_2022_22861_MOESM3_ESM.docx]

|  |  | Smile | | | | | Follow things with eyes | | | | | | | | | React to sound stimuli | | | | | | | | | | | | | Say no with head | | | | | | | | | | | | | | |  |
| --- | --- | --- | --- | --- | --- | --- | --- | --- | --- | --- | --- | --- | --- | --- | --- | --- | --- | --- | --- | --- | --- | --- | --- | --- | --- | --- | --- | --- | --- | --- | --- | --- | --- | --- | --- | --- | --- | --- | --- | --- | --- | --- | --- | --- |
|  |  | n | HR | CI | *p* | n | | | HR | | CI | | *p* | | | | n | | HR | | | CI | | | *p* | | | n | | | | HR | | | | CI | | | | *p* | | | |  |
| Gender | Male | 2826 | 1 | - | 0.1623 | 2685 | | | 1 | | - | | 0.1658 | | | | 6458 | | 1 | | | - | | | 0.7665 | | | 2902 | | | | 1 | | | | - | | | | 0.6907 | | | |  |
|  | Female |  | 1.05 | (0.98 - 1.13) |  |  | | | 1.05 | | (0.98 - 1.14) | |  | | | |  | | 0.99 | | | (0.95 - 1.04) | | |  | | |  | | | | 0.99 | | | | (0.92 - 1.06) | | | |  | | | |  |
| Province | Phnom Penh | 2826 |  | - | 0.1284 | 2685 | | | 1 | | - | | 0.3170 | | | | 6458 | | 1 | | | - | | | 0.9786 | | | 2902 | | | |  | | | |  | | | | 0.2936 | | | |  |
|  | Kratie |  | 1.07 | (0.98 - 1.17) |  |  | | | 1.05 | | (0.96 - 1.15) | |  | | | |  | | 1.00 | | | (0.94 - 1.06) | | |  | | |  | | | | 1.05 | | | | (0.96 - 1.14) | | | |  | | | |  |
|  | Ratanakiri |  | 1.11 | (1.01 - 1.22) |  |  | | | 1.08 | | (0.98 - 1.19) | |  | | | |  | | 0.99 | | | (0.93 - 1.06) | | |  | | |  | | | | 0.91 | | | | (0.84 - 1.00) | | | |  | | | |  |
| Mother education | No education | 2735 | 1 | - | 0.0030 | 2600 | | | 1 | | - | | 0.0017 | | | | 6013 | | 1 | | | - | | | 0.0042 | | | 2790 | | | | 1 | | | | - | | | | 0.0002 | | | |  |
|  | Primary |  | 1.17 | (1.05 - 1.29) |  |  | | | 1.18 | | (1.06 - 1.31) | |  | | | |  | | 1.11 | | | (1.03 - 1.18) | | |  | | |  | | | | 1.21 | | | | (1.09 - 1.33) | | | |  | | | |  |
|  | Secondary |  | 1.19 | (1.06 - 1.33) |  |  | | | 1.21 | | (1.08 - 1.36) | |  | | | |  | | 1.23 | | | (1.14 - 1.33) | | |  | | |  | | | | 1.31 | | | | (1.17 - 1.46) | | | |  | | | |  |
|  | High School/  University |  | 1.27 | (1.11 - 1.46) |  |  | | | 1.31 | | (1.15 - 1.51) | |  | | | |  | | 1.16 | | | (1.05 - 1.27) | | |  | | |  | | | | 1.22 | | | | (1.07 - 1.39) | | | |  | | | |  |
| Economic level | Poor | 2704 | 1 | - | 0.4899 | 2594 | | | 1 | | - | | 0.8423 | | | | 4384 | | 1 | | | - | | | 0.8110 | | | 2840 | | | | 1 | | | | - | | | | 0.0169 | | | |  |
|  | Median |  | 0.97 | (0.88 - 1.06) |  |  | | | 1.01 | | (0.92 - 1.11) | |  | | | |  | | 0.99 | | | (0.92 - 1.06) | | |  | | |  | | | | 1.11 | | | | (1.02 - 1.21) | | | |  | | | |  |
|  | Wealthy |  | 1.25 | (1.14 - 1.38) |  |  | | | 1.22 | | (1.11 - 1.35) | |  | | | |  | | 1.34 | | | (1.25 - 1.45) | | |  | | |  | | | | 1.17 | | | | (1.06 - 1.30) | | | |  | | | |  |
|  |  |  |  |  |  |  | | |  | |  | |  | | | |  | |  | | |  | | |  | | |  | | | |  | | | |  | | | |  | | | |  |
|  |  | Follow simple instructions | | | | | Interaction with others | | | | | | | | | | | Say few words | | | | | | | | | | |  | | | |  | | | |  | | | |  | | |  |
|  |  | n | HR | CI | *p* | | | n | | HR | | CI | | *p* | | | | n | | HR | | | CI | | | *p* | | | |  | | | |  | | | |  | | | |  | |  |
| Gender | Male | 2706 | 1 | - | 0.2799 | | | 5094 | | 1 | | - | | | 0.7358 | | | 4850 | | | 1 | | | - | | | 0.4026 | | | |  | | | |  | | | |  | | | |  | |
|  | Female |  | 0.96 | (0.89 - 1.03) |  | | |  | | 1.01 | | (0.96 - 1.07) | | |  | | |  | | | 1.02 | | | (0.97 - 1.08) | | |  | | | |  | | | |  | | | |  | | | |  | |
| Province | Phnom Penh | 2706 | 1 | - | < 0.0001 | | | 5094 | |  | | - | | | < 0.0001 | | | 4850 | | |  | | | - | | | 0.0001 | | | |  | | | |  | | | |  | | | |  | |
|  | Kratie |  | 0.75 | (0.69 - 0.82) |  | | |  | | 1.26 | | (1.17 - 1.35) | | |  | | |  | | | 1.15 | | | (1.07 - 1.24) | | |  | | | |  | | | |  | | | |  | | | |  | |
|  | Ratanakiri |  | 0.88 | (0.81 - 0.97) |  | | |  | | 1.28 | | (1.19 - 1.38) | | |  | | |  | | | 1.26 | | | (1.16 - 1.36) | | |  | | | |  | | | |  | | | |  | | | |  | |
| Mother education | No education | 2603 | 1 | - | 0.0689 | | | 4770 | | 1 | | - | | | 0.0469 | | | 4546 | | | 1 | | | - | | | 0.0111 | | | |  | | | |  | | | |  | | | |  | |
|  | Primary |  | 1.10 | (0.99 - 1.22) |  | | |  | | 1.08 | | (1.00 - 1.16) | | |  | | |  | | | 1.11 | | | (1.02 - 1.19) | | |  | | | |  | | | |  | | | |  | | | |  | |
|  | Secondary |  | 1.28 | (1.14 - 1.44) |  | | |  | | 1.19 | | (1.09 - 1.30) | | |  | | |  | | | 1.20 | | | (1.10 - 1.31) | | |  | | | |  | | | |  | | | |  | | | |  | |
|  | High School/  University |  | 1.21 | (1.06 - 1.38) |  | | |  | | 1.10 | | (0.99 - 1.22) | | |  | | |  | | | 1.13 | | | (1.01 - 1.26) | | |  | | | |  | | | |  | | | |  | | | |  | |
| Economic level | Poor | 2646 | 1 | - | 0.0004 | | | 3589 | | 1 | | - | | | 0.4388 | | | 3578 | | | 1 | | | - | | | 0.1797 | | | |  | | | |  | | | |  | | | |  | |
|  | Median |  | 1.17 | (1.07 - 1.28) |  | | |  | | 1.03 | | (0.95 - 1.12) | | |  | | |  | | | 1.06 | | | (0.98 - 1.14) | | |  | | | |  | | | |  | | | |  | | | |  | |
|  | Wealthy |  | 1.23 | (1.11 - 1.37) |  | | |  | | 1.49 | | (1.38 - 1.62) | | |  | | |  | | | 1.45 | | | (1.34 - 1.58) | | |  | | | |  | | | |  | | | |  | | | |  | |

HR: Hazard ratio, CI: Confidence Interval

**Supplementary table 3**: Crude associations between the ages for achieving cognitive milestones and sociodemographic and economic characteristics of children
