# Supplementary material for: The associations between stunting and wasting at 12 months of age and developmental milestones delays in a cohort of Cambodian children
Source: Sci Rep. 2022 Oct 25;12:17859. doi: 10.1038/s41598-022-22861-2 (PMC9596435; doi:10.1038/s41598-022-22861-2)
Supplement: Supplementary file 4 — Supplementary Table 4. [file 41598_2022_22861_MOESM4_ESM.docx]

|  | Crude associations | | | | Adjusted associations (model 1^1^) | | | | Adjusted associations (model 2 ^2^) | | | |
| --- | --- | --- | --- | --- | --- | --- | --- | --- | --- | --- | --- | --- |
|  | n | HR | CI | *p* | n | HR | CI | *p* | n | HR | CI | *p* |
| Motor milestones |  |  |  |  |  |  |  |  |  |  |  |  |
| Bring things to mouth | 1856 | 0.82 | (0.74 - 0.92) | 0.0004 | 1809 | 0.78 | (0.70 - 0.87) | < 0.0001 | 1780 | 0.79 | (0.70 - 0.88) | < 0.0001 |
| Sitting | 3074 | 0.84 | (0.77 - 0.91) | < 0.0001 | 2913 | 0.84 | (0.77 - 0.92) | 0.0001 | 2216 | 0.78 | (0.71 - 0.87) | < 0.0001 |
| Eat with hands | 2808 | 0.90 | (0.83 - 0.98) | 0.0185 | 2654 | 0.86 | (0.79 - 0.95) | 0.0013 | 1971 | 0.84 | (0.76 - 0.94) | 0.0014 |
| Standing | 3019 | 0.77 | (0.71 - 0.84) | < 0.0001 | 2864 | 0.81 | (0.74 - 0.89) | < 0.0001 | 2183 | 0.78 | (0.71 - 0.87) | < 0.0001 |
| Walking | 2766 | 0.78 | (0.72 - 0.86) | < 0.0001 | 2631 | 0.77 | (0.70 - 0.84) | < 0.0001 | 2040 | 0.74 | (0.67 - 0.83) | < 0.0001 |
| Palmer grasp | 2594 | 0.93 | (0.85 - 1.01) | 0.0987 | 2459 | 0.89 | (0.81 - 0.98) | 0.0138 | 1898 | 0.89 | (0.80 - 1.00) | 0.0384 |
| Drink from a cup | 2816 | 0.90 | (0.83 - 0.98) | 0.0193 | 2675 | 0.88 | (0.80 - 0.96) | 0.0054 | 2072 | 0.90 | (0.81 - 1.00) | 0.0423 |
|  |  |  |  |  |  |  |  |  |  |  |  |  |
| Cognitive milestones |  |  |  |  |  |  |  |  |  |  |  |  |
| Smile | 2004 | 0.91 | (0.82 - 1.01) | 0.0624 | 1950 | 0.89 | (0.80 - 0.99) | 0.0304 | 1886 | 0.91 | (0.81 - 1.01) | 0.0847 |
| Follow things with eyes | 1930 | 0.89 | (0.80 - 0.99) | 0.0255 | 1878 | 0.88 | (0.79 - 0.98) | 0.0197 | 1826 | 0.90 | (0.80 - 1.01) | 0.0690 |
| React to sound stimuli | 3051 | 0.86 | (0.79 - 0.94) | 0.0004 | 2888 | 0.86 | (0.78 - 0.93) | 0.0004 | 2191 | 0.84 | (0.76 - 0.93) | 0.0005 |
| Say no with head | 1010 | 0.88 | (0.76 - 1.02) | 0.0986 | 981 | 0.87 | (0.74 - 1.02) | 0.0737 | 962 | 0.85 | (0.73 - 0.99) | 0.0403 |
| Follow simple instruction | 967 | 0.74 | (0.64 - 0.87) | 0.0002 | 939 | 0.76 | (0.65 - 0.90) | 0.0008 | 921 | 0.72 | (0.61 - 0.85) | 0.0001 |
| Interaction with others | 2648 | 0.89 | (0.82 - 0.97) | 0.0116 | 2506 | 0.86 | (0.78 - 0.94) | 0.0012 | 1856 | 0.86 | (0.77 - 0.96) | 0.0060 |
| Say few words | 2558 | 0.91 | (0.84 - 1.00) | 0.0526 | 2433 | 0.88 | (0.80 - 0.97) | 0.0072 | 1845 | 0.89 | (0.80 - 0.99) | 0.0280 |

HR: Hazard ratio, CI: Confidence Interval

^1^ Model 1: associations with stunting controlling for province, mother education and child gender

^2^ Model 2: associations with stunting controlling for province, mother education, child gender and economic tertile

**Supplementary table 4**: Crude and adjusted associations between the ages for achieving motor and cognitive milestones and stunting at 12 months
