# Supplementary material for: The associations between stunting and wasting at 12 months of age and developmental milestones delays in a cohort of Cambodian children
Source: Sci Rep. 2022 Oct 25;12:17859. doi: 10.1038/s41598-022-22861-2 (PMC9596435; doi:10.1038/s41598-022-22861-2)
Supplement: Supplementary file 5 — Supplementary Table 5. [file 41598_2022_22861_MOESM5_ESM.docx]

|  | Crude associations | | | | Adjusted associations (model 1^1^) | | | | Adjusted associations (model 2 ^2^) | | | |
| --- | --- | --- | --- | --- | --- | --- | --- | --- | --- | --- | --- | --- |
|  | n | HR | CI | *p* | n | HR | CI | *p* | n | HR | CI | *p* |
| Motor milestones |  |  |  |  |  |  |  |  |  |  |  |  |
| Bring things to mouth | 1859 | 0.73 | (0.63 - 0.84) | < 0.0001 | 1812 | 0.72 | (0.62 - 0.83) | < 0.0001 | 1783 | 0.71 | (0.61 - 0.82) | < 0.0001 |
| Sitting | 3077 | 0.96 | (0.85 - 1.08) | 0.4891 | 2916 | 0.94 | (0.83 - 1.06) | 0.319 | 2219 | 0.79 | (0.69 - 0.91) | 0.0011 |
| Eat with hands | 2811 | 0.66 | (0.58 - 0.75) | < 0.0001 | 2657 | 0.65 | (0.57 - 0.73) | < 0.0001 | 1974 | 0.64 | (0.55 - 0.74) | < 0.0001 |
| Standing | 3021 | 0.93 | (0.82 - 1.05) | 0.219 | 2866 | 0.95 | (0.84 - 1.08) | 0.4274 | 2185 | 0.89 | (0.77 - 1.02) | 0.0906 |
| Walking | 2766 | 0.85 | (0.75 - 0.97) | 0.015 | 2631 | 0.86 | (0.75 - 0.98) | 0.0209 | 2041 | 0.85 | (0.73 - 0.98) | 0.0215 |
| Palmer grasp | 2595 | 0.67 | (0.59 - 0.76) | < 0.0001 | 2460 | 0.64 | (0.56 - 0.73) | < 0.0001 | 1899 | 0.63 | (0.54 - 0.73) | < 0.0001 |
| Drink from a cup | 2818 | 0.94 | (0.82 - 1.06) | 0.2938 | 2677 | 0.92 | (0.81 - 1.05) | 0.1939 | 2074 | 0.89 | (0.77 - 1.03) | 0.1244 |
|  |  |  |  |  |  |  |  |  |  |  |  |  |
| Cognitive milestones |  |  |  |  |  |  |  |  |  |  |  |  |
| Smile | 2004 | 0.75 | (0.65 - 0.87) | 0.0001 | 1950 | 0.73 | (0.63 - 0.85) | < 0.0001 | 1886 | 0.74 | (0.63 - 0.86) | 0.0001 |
| Follow things with eyes | 1932 | 0.75 | (0.65 - 0.88) | 0.0003 | 1880 | 0.74 | (0.63 - 0.86) | < 0.0001 | 1828 | 0.74 | (0.64 - 0.87) | 0.0001 |
| React to sound stimuli | 3055 | 0.7 | (0.62 - 0.79) | < 0.0001 | 2892 | 0.69 | (0.61 - 0.78) | < 0.0001 | 2195 | 0.63 | (0.55 - 0.72) | < 0.0001 |
| Say no with head | 1013 | 0.89 | (0.74 - 1.07) | 0.2283 | 984 | 0.91 | (0.75 - 1.10) | 0.316 | 965 | 0.90 | (0.74 - 1.09) | 0.2659 |
| Follow simple instruction | 970 | 0.72 | (0.60 - 0.87) | 0.0008 | 942 | 0.76 | (0.63 - 0.93) | 0.0049 | 924 | 0.74 | (0.61 - 0.90) | 0.0022 |
| Interaction with others | 2650 | 0.67 | (0.59 - 0.76) | < 0.0001 | 2508 | 0.64 | (0.56 - 0.73) | < 0.0001 | 1857 | 0.63 | (0.54 - 0.73) | < 0.0001 |
| Say few words | 2559 | 0.69 | (0.61 - 0.79) | < 0.0001 | 2434 | 0.68 | (0.60 - 0.78) | < 0.0001 | 1847 | 0.66 | (0.57 - 0.77) | < 0.0001 |

HR: Hazard ratio, CI: Confidence Interval

^1^ Model 1: associations with wasting controlling for province, mother education and child gender

^2^ Model 2: associations with wasting controlling for province, mother education, child gender and economic tertile

**Supplementary table 5**: Crude and adjusted associations between the ages for achieving motor and cognitive milestones and wasting at 12 months
